# Electroacupuncture Reduces Visceral Pain Via Cannabinoid CB2 Receptors in a Mouse Model of Inflammatory Bowel Disease

**DOI:** 10.3389/fphar.2022.861799

**Published:** 2022-03-25

**Authors:** Hong Zhang, Wei He, Xue-Fei Hu, Yan-Zhen Li, Yong-Min Liu, Wen-Qiang Ge, Ou-Yang Zhanmu, Chao Chen, Yu-Ye Lan, Yang-Shuai Su, Xiang-Hong Jing, Bing Zhu, Hui-Lin Pan, Ling-Ling Yu, Man Li

**Affiliations:** ^1^ Department of Neurobiology, School of Basic Medicine, Tongji Medical College, Huazhong University of Science and Technology, Wuhan, China; ^2^ Research Center of Meridians, Institute of Acupuncture and Moxibustion, China Academy of Chinese Medical Sciences, Beijing, China; ^3^ Department of Anesthesiology and Perioperative Medicine, The University of Texas MD Anderson Cancer Center, Houston, TX, United States; ^4^ Institute of Integrated Traditional Chinese and Western Medicine, Tongji Hospital, Tongji Medical College, Huazhong University of Science and Technology, Wuhan, China

**Keywords:** electroacupunture, inflammatory bowel disease, CB2 receptor, visceral pain, macrophage

## Abstract

Inflammatory bowel disease (IBD) results in chronic abdominal pain in patients due to the presence of inflammatory responses in the colon. Electroacupuncture (EA) is effective in alleviating visceral pain and colonic inflammation associated with IBD. Cannabinoid CB2 receptor agonists also reduce colonic inflammation in a mouse model of IBD. However, whether EA reduces visceral pain and colonic inflammation *via* the CB2 receptor remains unknown. Here, we determined the mechanism of the antinociceptive effect of EA in a mouse model of IBD induced by rectal perfusion of 2,4,6-trinitrobenzenesulfonic acid solution (TNBS). EA or sham EA was performed at the bilateral Dachangshu (BL25) point for seven consecutive days. The von Frey and colorectal distension tests were performed to measure mechanical referred pain and visceral pain. Western blotting and immunohistochemistry assays were carried out to determine the expression of IL-1β and iNOS and activation of macrophages in the colon tissues. We found that EA, but not sham EA, attenuated visceral hypersensitivity and promoted activation of CB2 receptors, which in turn inhibited macrophage activation and the expression of IL-1β and iNOS. The effects of EA were blocked by AM630, a specific CB2 receptor antagonist, and by CB2 receptor knockout. Our findings suggest that EA attenuates mechanical allodynia and visceral hypersensitivity associated with IBD by activating CB2 receptors and subsequent inhibition of macrophage activation and expression of IL-1β and iNOS.

## Perspective

This study presents new evidence about CB2 receptors in the antinociceptive effect of EA in a mouse model of IBD. This work helps the clinicians to understand how EA reduces visceral pain associated with IBD.

## Introduction

Inflammatory bowel disease (IBD) is caused by inflammation in the gastrointestinal tract and is accompanied by chronic abdominal pain, diarrhea, bloody stools, anemia, and weight loss. Crohn’s disease and ulcerative colitis represent two forms of IBD and are characterized by uncontrolled activation of the host’s intestinal immune cells (Neurath, 2019). The recurrent nature of the inflammatory process in IBD highlights the clinical need for effective anti-inflammatory therapies. However, current therapies have limited efficacy and produce serious side effects. Therefore, there is an urgent need for effective and safety anti-inflammatory treatment options for IBD patients.

EA has been widely used to treat many acute and chronic diseases and is effective for the gastrointestinal system disorders, particularly IBD (Wang et al., 2020). The antinociceptive effect of EA on IBD may involve anti-inflammatory effect *via* the cholinergic reflex (Borovikova et al., 2000) and the hypothalamic-pituitary-adrenal axis (Wei et al., 2019). Other mechanisms may include the opioid system (Kim et al., 2005), the adenosine pathway (Song et al., 2019), and macrophage polarization (Song et al., 2019). It is unclear whether these mechanisms mediate the effect of EA on the inflammatory response in IBD.

The cannabinoid system, which has a long history as a treatment for symptoms associated with gastrointestinal disorders, is involved in the control of tissue homeostasis and important gut functions such as motor and sensory activity, nausea, vomiting, maintenance of epithelial barrier integrity and proper cellular micro-environment (Yuce et al., 2010; Sharkey et al., 2014; Garcia-Arencibia et al., 2019; Scheau et al., 2021). Cannabis or its components act through CB1 and CB2 receptors, which are found throughout the GI system (e.g., liver, pancreas, stomach, small and large intestine) (Naftali et al., 2014). CB2 receptors mainly exist in immune cells, such as plasma cells and macrophages (Wright et al., 2005). In recent years, the role of CB2 receptors in colitis has been increasingly recognized. Administration of the CB2 receptor agonist AM1241 reduces colonic inflammation in a mouse model of TNBS-induced colitis (Storr et al., 2009). This leads to a reasonable speculation that the activation of CB2 receptors may contribute to the amelioration of colitis. Thus, targeting the CB2 receptor may constitute an alternative approach to treat IBD, because this receptor is mainly expressed in immune cells.

In this study, we determined whether EA attenuates mechanical allodynia, visceral hyperalgesia, and intestinal inflammation by activating CB2 receptors in TNBS-treated IBD in mice. Our study suggest that EA reduces pain symptoms in IBD by reducing intestinal inflammation *via* CB2 receptor activation. This new information provides a rationale for using EA to treat visceral pain associated with IBD.

## Materials and Methods

### Animals

Adult male C57 BL/6 mice (8-week-old; 20–25 g, *n* = 6 mice/group) were purchased from Experimental Animal Center of Tongji Medical College of Huazhong University of Science and Technology. Twenty-four CB2 receptor knockout mice were kindly provided by Dr. Nancy E. Buckley (National Institutes of Health, Bethesda, United States). Animals were individually housed in cages at 22 ± 2°C and a 12 h light/dark cycle and had free access to food and water. All animal procedures were approved by the Institutional Animal Care and Use Committee at Huazhong University of Science and Technology and conformed to the ethical guidelines of the International Association for the Study of Pain (IASP).

### IBD Model

**FIGURE 1:**
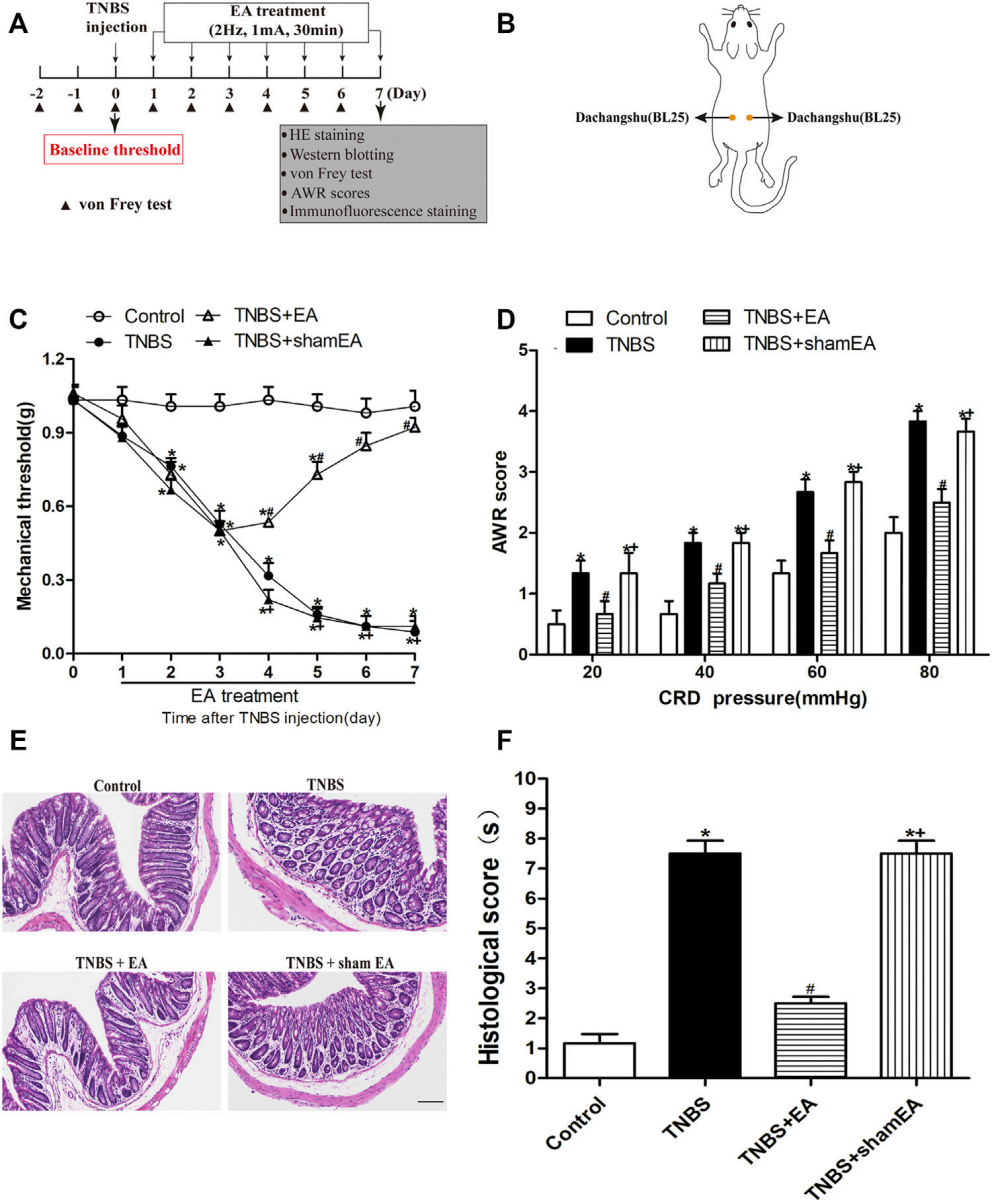
EA attenuates TNBS-induced mechanical allodynia, visceral hyperalgesia and inflammatory in the intestine. **(A)** Experimental flowchart. **(B)** Schematic show the lumbodorsal BL25 acupoint. **(C)** Time course of the effect of BL25 EA on mechanical withdrawal threshold of the hind paws of TNBS-treated mice. **(D)** AWR scores of vehicle-treated and TNBS-treated mice on day 7 after vehicle or TNBS treatment. **(E)** Representative H&E staining of the colon tissue sections. Scale bar, 200 µm. **(F)** Histological score in each group (*n* = 6 per group). *p < 0.05 versus with Control group, #*p* < 0.05 versus with TNBS group, +*p* < 0.05 versus with TNBS + EA group.

The IBD model was induced by slow intra-rectal instillation of TNBS (Sigma-Aldrich, St. Louis, MO, United States) solution (50 μl of 5% w/v TNBS mixed with 50 μl absolute ethanol) into the lumen of the colon in anesthetized mice, as previously reported (Hou et al., 2019). An experimental design timeline is presented in Figure 1A.

### Nociceptive Behavioral Tests

Animal behavior tests were performed from 9:00 a.m. to 12:00 p.m. Mice were first habituated to the test environment for 30 min. The mechanical thresholds were tested for 3 days before TNBS injection and the average value of the 3-day tests was calculated as the baseline threshold. After TNBS injection, the nociceptive thresholds were tested once a day for seven consecutive days. Then a series of calibrated von Frey filaments (0.02, 0.04, 0.07, 0.16, 0.4, 0.6, 1.0 and 1.4 g, Wood Dale, United States) were applied perpendicularly to the mid-plantar surface of the left hind paw to bend the filament for 6s. The mechanical threshold of mice was measured by using the “up and down” method (Chaplan et al., 1994) and the test of withdrawal threshold was repeated two times in each mouse, and the mean value was calculated. Paw withdrawal or licking feet was considered as a pain-like response.

Visceral hyperalgesia measurements were made on the seventh day after TNBS injection. The colorectal distension (CRD) method was used to observe the abdominal withdrawal reflex scores (AWRs) of mice to assess visceral hyperalgesia. The CRD test was performed in a step-by-step compression mode (20/40/60/80 mmHg). Each pressure value was measured twice, each test lasting 30 s, with an interval of 4 min, AWRs were recorded and averaged. The scoring criteria were the same as the Al-Chaer’s method (Bao et al., 2019): no behavioral response to CRD was rated as 0, short pauses in head or body movements during stimulation was rated as 1; abdominal muscle contraction during stimulation was rated as 2; abdominal lifting was rated as 3; and body arch, pelvic cavity or scrotum lifting was rated as 4.

### EA Treatment

EA treatment started on day 1 after TNBS injection. Before EA, mice were gently immobilized using homemade clothes (10 × 10 cm) and their limbs were pulled out through holes in the clothes. A pair of stainless steel acupuncture needles (0.25 mm × 13 mm, Beijing Zhongyan Taihe Medical Instruments Co., Ltd., China) were inserted into a depth of 4 mm into bilateral sides of Dachangshu (BL25, the waist of the fourth lumbar vertebra at about 7 mm bilaterally to midline) acupoint (Wang et al., 2015). The handles of these needles were connected to Han’s Acupoint Nerve Stimulator (LH202, Huawei Co., Ltd., Beijing, China) with a frequency of 2 Hz and intensity of 1 mA for 30 min, once per day for seven consecutive days. In this study, for sham EA group, real acupuncture needles were shallowly inserted into nonacupoint locations (0.3–0.5 mm), so as to reduce the physiological effect of sham acupuncture group. The animals remained awake and motionless during the treatment and showed no evident signs of distress. The control group and TNBS group were only manipulated with self-made clothes without other treatment.

### Drug Administration

The CB2 receptor inhibitor AM630 (Sigma-Aldrich, St. Louis, United States) was dissolved in a vehicle consisting of 1:2:7 ratio of dimethylsulfoxide (DMSO), Tween 80 and normal saline as previously described (Liu et al., 2021). Mice were intraperitoneally administrated with 100 μl of AM630 at 5 mg/kg body weight (Lopes et al., 2020) or vehicle 30 min before EA treatment, every day for 7 days.

### Experimental Design

To test the EA’s effect, C57BL/6 mice were randomly divided into Control (vehicle of TNBS), TNBS, TNBS plus EA (TNBS + EA) or TNBS plus sham EA (TNBS + sham EA) groups. To determine the role of CB2 receptor in EA in the treatment of IBD-associated visceral hypersensitivity and inflammation, C57BL/6 mice were randomly divided into Control + DMSO (vehicle of AM630, sterilized DMSO), TNBS + DMSO, TNBS + EA + DMSO, TNBS + AM630 + EA groups; CB2 receptor knockout mice were randomly divided into Control (vehicle of TNBS), TNBS, TNBS plus EA (TNBS + EA) or TNBS plus sham EA (TNBS + sham EA) groups.

### Western Blotting

Mice were anaesthetized with 3% isoflurane, and their colon tissues were immediately excised. Tissues were initially placed on ice and stored at −80°C, pending protein extraction. The tissues were then lysed by adding 40 mg/ml RIPA lysis buffer (Biosharp, China) and 40 mg/ml phenylmethyl sulfonyl fluoride (Biosharp, China) to the samples for 30 min. The lysate was collected and centrifuged at 12,000 rpm for 15 min at 4°C, and the protein contents were quantified by using the Enhanced BCA Protein Assay Kit (Beyotime Biotechnology, China). The protein (40 mg) was denatured in loading buffer at 95°C for 5 min, separated on a 10%/12% glycine-SDS-PAGE gel (10%: CB2 receptor, iNOS; 12%: IL-1β) (Beyotime Biotechnology, China), and then transferred onto a PVDF membrane (Millipore Immobilon-P, United States). The membranes were blocked with 5% non-fat milk at room temperature for 1 h, followed by primary antibodies at 4°C overnight: anti-CB2 receptor rabbit antibody (diluted 1/500; Abcam), anti-Turblin mouse antibody (diluted 1/2000; Santa Cruz Technology), anti-iNOS rabbit antibody (diluted 1/1000; Abcam), and anti-IL-1β rabbit antibody (diluted 1/1000, Abcam). Then, the membranes were incubated with IgG (diluted 1/20,000, Abcam). The signals were developed using Super Signal West Pico chemiluminescent substrate (Thermo Scientific, United States). The densitometric analysis of the protein band images was performed using ImageJ software (NIH, Bethesda, MD, United States).

### Histopathology Assessment

For histological examination, the colonic tissue was dehydrated with 4% paraformaldehyde and then paraffin embedded. Tissue sections (4 μm) were de-paffinized in xylene and stained with hematoxylin and eosin (H&E) to assess intestinal inflammation. H&E staining was scored by a blinded observer using a previously described method (Kim et al., 2012): crypt architecture (normal is 0 point, loss of crypts is 3 point), inflammatory cell infiltration (normal is 0 point, dense inflammatory infiltrate is 3 point), muscle thickening (base of crypt sits on the muscularis mucosae is 0 point, marked muscle thickening present is 3 point), goblet cell depletion (absent is 0 point, present is 3 point) and crypt abscess (absent is 0 point, present is one point).

### Immunofluorescence Labeling

Mice were deeply anesthetized by intraperitoneal injection of 6% chloral hydrate. The heart was perfused with 0.9% normal saline (40°C) and then fixed with 4% paraformaldehyde (4°C) for 12 h. Colon tissues were dehydrated in 20% sucrose solution (12 h) and 30% sucrose solution (12 h) prepared with 0.1 M PBS buffer. Transverse section of the colon was cut to 15 μm thick using a cryostat. The sections were pasted on gelatin-coated glass slides and dried overnight. The slides were washed in 0.01 M PBS for four times in PBS for 5 min, and blocked for 1 h with 5% donkey serum and 0.3% triton X100 in PBS and then incubated with the following primary antibodies at 4°C overnight: mouse anti-CD68 (1:200; Abcam, Cambrige, United Kingdom, #ab955) for identification of macrophages, rabbit anti-iNOS (1:200; Abcam, #ab178945) and rabbit anti-CB2 receptor (1:200; Abcam, #ab3561). Subsequently, sections were rinsed four times in PBS for 5 min and incubated with corresponding secondary antibodies at room temperature for 1 h: donkey anti-mouse IgG conjugated with Dylight 488 (1:400) or donkey anti-rabbit IgG conjugated with Dylight 594 (1:400). The slides were washed 4 times with 0.01 M PBST for 5 min each time. Sections were washed three times in PBS and then incubated with Hoechst for the nucleus staining for 5 min. Lastly, sections were washed three times in PBS for 5 min and then cover-slipped. The tissue sections were viewed under a laser confocal microscope (FV500-IX7, Olympus, Japan). The images were analyzed using ImageJ software (NIH, Bethesda, United States).

### Statistical Analysis

The results were presented as the mean ± SEM. Two-way repeated measures ANOVA followed by Bonferroni’s *post hoc* test was used to compare the mechanical pain threshold and AWRs in each group. One-way ANOVA and Newman-Keuls *post hoc* test were used in the analysis of biochemical data. SPSS 23.0 was used for the data analysis. *p* value of less than 0.05 was considered statistically significant.

## Results

### EA Alleviates Visceral Hyperalgesia, Mechanical Allodynia and Intestinal Pathology Induced by TNBS

We first established a mouse model of TNBS-induced colitis. In the von Frey test, a measure of referred pain, the withdrawal threshold was significantly decreased at the second day after TNBS injection, and this effect lasted at least 7 days (*p* < 0.05, Figure 1C). In the CRD test, TNBS-treated mice exhibited a significant decrease in AWRs (*p* < 0.05, Figure 1D).

To examine the role of EA on TNBS-induced nociceptive responses, EA was performed at BL25 acupoints for 30 min on days 1–7 after TNBS injection. EA, but not sham EA, reduced mechanical allodynia measured on days 4–7 and visceral hyperalgesia measured on the seventh day. No significant differences in behavioral responses were observed at any time among C57BL/6 mice treated with Control or Control + EA (*p >* 0.05, Figures 1C,D).

As shown in Figure 1E, the TNBS group clearly exhibited an acute inflammatory response with mucosal erosion, congestion, crypt reduction, neutrophil cell infiltrates and goblet cells disrupted destruction compared to the Control group. Compared with the TNBS group, EA showed a significant improvement in mucosal inflammatory, but not by sham EA (Figure 1F). These results suggest that EA at BL25 is effective in reducing the nociceptive and inflammatory responses in the colon induced by TNBS.

### EA Inhibits the Expression of IL-1β and iNOS in Inflamed Colon Tissues of TNBS-Treated Mice

**FIGURE 2:**
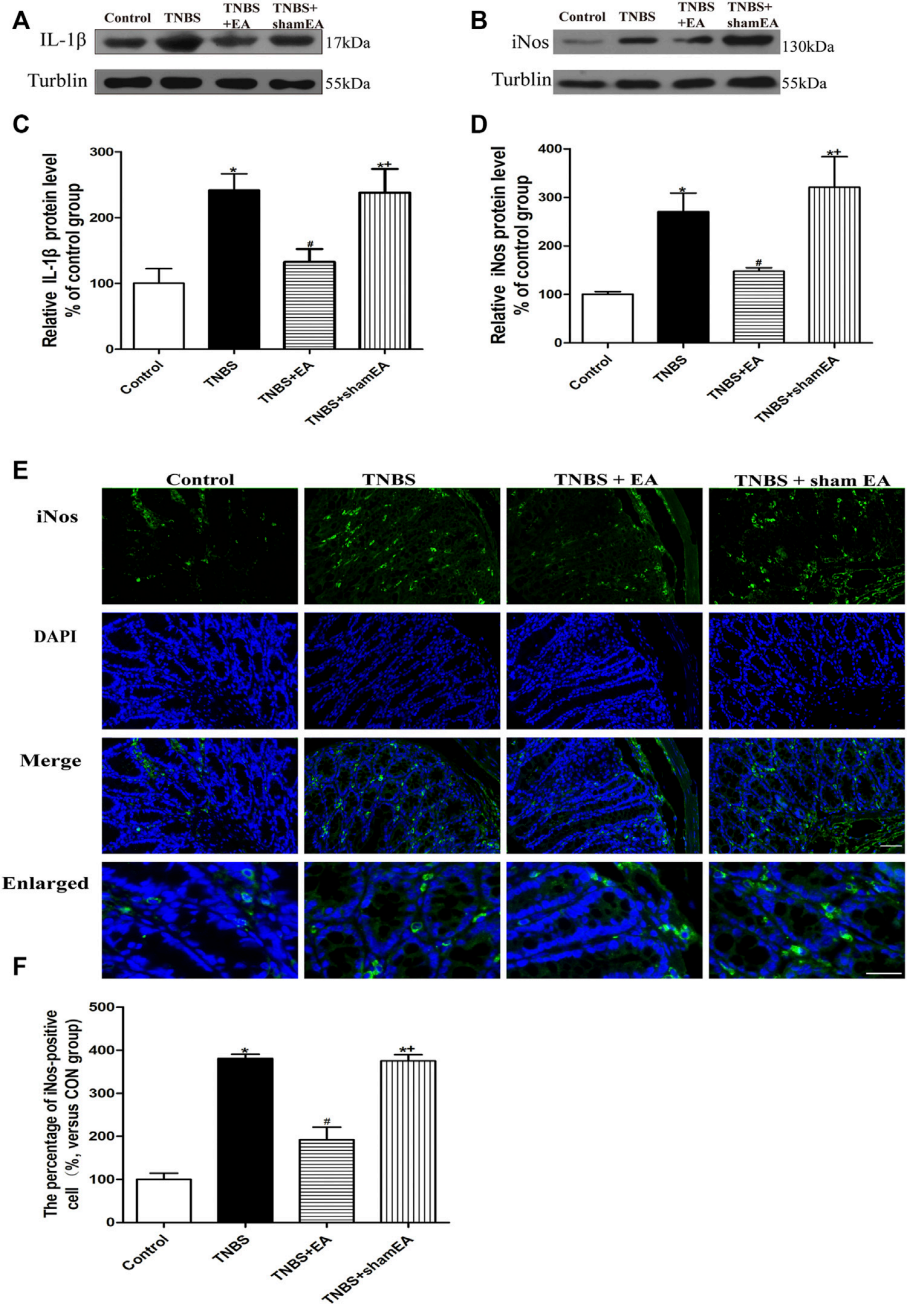
EA inhibits inflammatory responses in TNBS-treated mice. **(A**–**E)** Representative western blot images show the protein levels of IL-1β **(A)** and iNOS **(B)** in the colon tissues. Summary data show the relative protein level of IL-1β **(C)** and iNOS **(D)** in the colon tissues. **(E)** Representative images of immunofluorescence staining in which cell nuclei were labeled with DAPI (blue) and anti-iNOS antibody (green) in the colon tissues. **(F)** Summary data showing the number of iNOS-positive cells per observation field. Data are expressed as mean ± SEM (*n* = 6 per group). Scale bar, 50 μm *p < 0.05 versus with Control group, #*p* < 0.05 versus with TNBS group, +*p* < 0.05 versus with TNBS + EA group.

To determine the anti-inflammatory effect of EA, we examined the effect of EA on the expression level of IL-1β and iNOS in the colonic tissue of TNBS-treated mice. TNBS-treated mice significantly increased the protein levels of IL-1β and iNOS in colonic tissues compared with the Control group (*p* < 0.05, Figures 2A–D). EA significantly decreased the expression levels of IL-1β and iNOS compared with the TNBS group (*p* < 0.05, Figures 2A–D) or the sham EA group. Similarly, in the immunofluorescent assays, TNBS significantly elevated iNOS expression, and EA significantly decreased the iNOS expression (*p* < 0.05, Figures 2E,F). These findings indicated that EA attenautes the colonic inflammatory response by inhibiting the expression of pro-inflammatory cytokines such as IL-1β and iNOS.

### EA Increases CB2 Receptor Expression and Attenuates Macrophages Activation in Inflamed Colon Tissues of TNBS-Treated Mice

**FIGURE 3:**
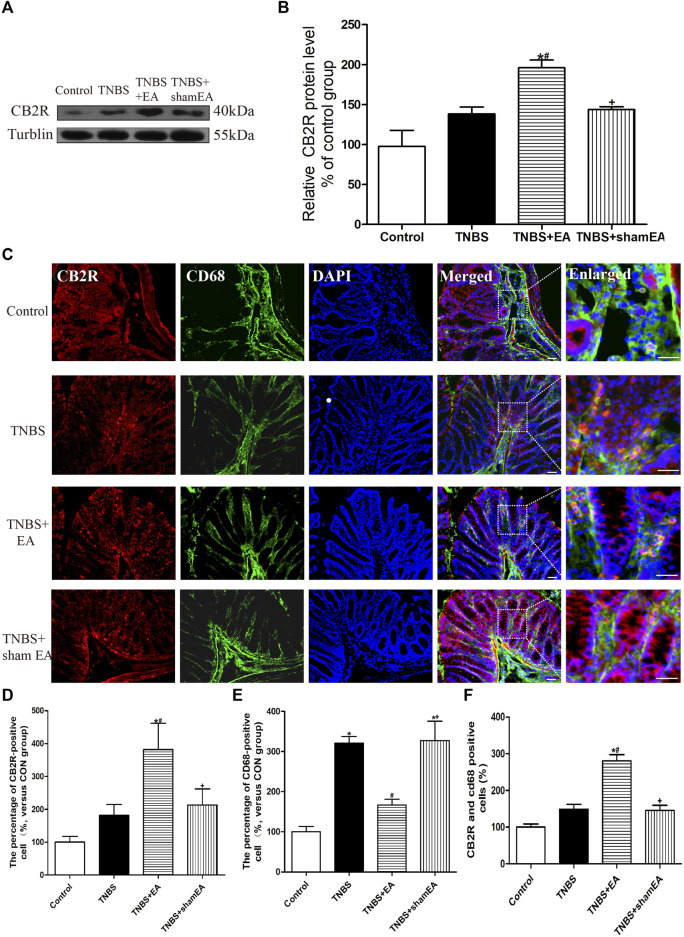
EA increased CB2 receptor activation in macrophages in the colon tissues. **(A)** Representative western blot images show the protein level of CB2 receptors in the colon tissues. **(B)** Summary data show the relative protein level of CB2 receptors in the colon tissues. **(C)** Representative images of immunofluorescence staining using DAPI (blue), anti-CD68 antibody (macrophages) (green) and CB2 receptor antibody (red) in the colon tissues. Summary of the number of CB2 receptor- **(D)** and CD68^−^ **(E)** positive cells per observation field. **(F)** Summary data show the percentage of double-labeled cells in the colon tissues. Data are expressed as mean ± SEM (*n* = 6 per group). Merged, scale bar, 50 μm; Enlarged, scale bar, 50 μm *p < 0.05 versus with Control group, #*p* < 0.05 versus with TNBS group, +*p* < 0.05 versus with TNBS + EA group.

We have shown that the antinociceptive effect of EA on inflammatory pain is mediated by peripheral CB2 receptors and that CB2 receptors activation inhibits NLRP3 inflammasomes in macrophages *in vitro* (Gao et al., 2018). Next, we determined whether EA affects the intestinal CB2 receptor expression induced by TNBS. Compared with the Control group, TNBS significantly reduced the expression level of CB2 receptors in the colonic tissues (*p* < 0.05, Figures 3A,B). EA treatment significantly increased the protein level of CB2 receptors in the colonic tissues (*p* < 0.05, Figures 3A,B).

Double immunofluorescence labeling showed that CB2 receptors were colocalized with CD68, a macrophages marker. Comparable with the Control group, TNBS treatment increased CD68 labeling and decreased the number of colocalized CD68 and CB2 receptors (*p* < 0.05, Figures 3C–F). Comparable with the TNBS group, EA at BL25 inhibited the expression of CD68 and increased the number of colocalized CD68 and CB2 receptors (*p* < 0.05, Figures 3C–F). These data suggest that EA reduces macrophages activation and increases CB2 receptor expression in the inflamed colon tissues of TNBS-treated mice.

### Blocking CB2 Receptors Antagonizes the Antinociceptive and Anti-Inflammatory Effects of EA

**FIGURE 4:**
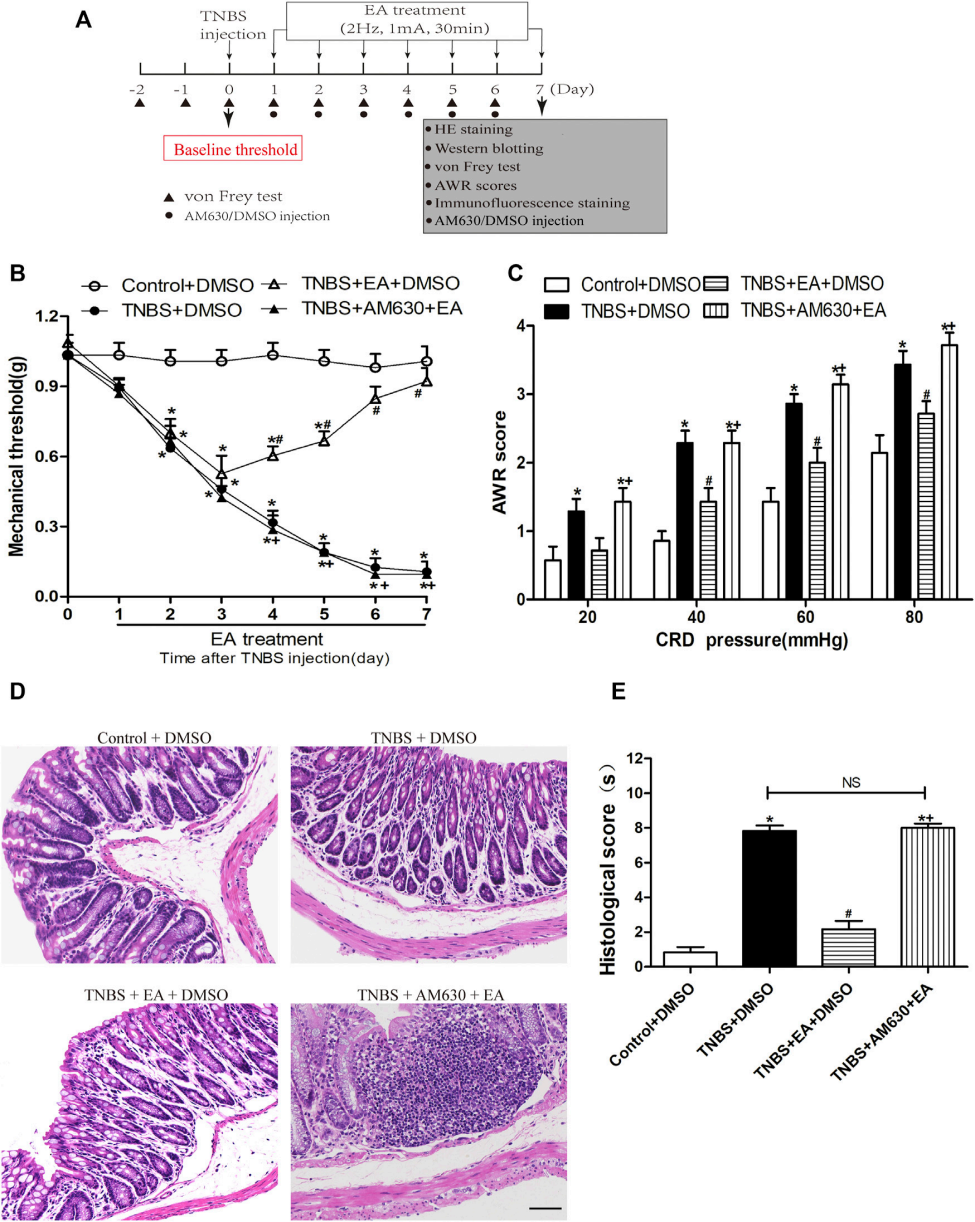
Pharmacological blockade of CB2 receptors reverses the EA’s effects on TNBS-induced visceral hypersensitivities and colonic morphology. **(A)** Experimental protocol show treatment schedule of AM630, the specific CB2 receptor antagonist. **(B)** Time course effect of AM630 treatment on the EA’s antinociceptive effect on TNBS-treated mice. **(C)** AWR scores of TNBS-treated mice on day 7. **(D)** Representative H&E staining of colon tissue sections. Scale bar, 200 µm. **(E)** Histological score in each group (*n* = 6 per group). *p < 0.05 versus with Control + DMSO group, #*p* < 0.05 versus with TNBS + DMSO group, +*p* < 0.05 versus with TNBS + EA + DMSO group. NS: no significance versus TNBS + EA + AM630 group as indicated.

To determine the role of CB2 receptors in EA’s antinociceptive and anti-inflammatory in TNBS-treated mice, wild-type (WT) mice received systemic delivery of AM630, a specific CB2 receptor antagonist 30 min before each EA treatment. The antinociceptive effects of EA were blocked by administration of AM630 (*p* > 0.05, Figures 4B,C). Compared with the Control + DMSO group, the TNBS + DMSO group and TNBS + AM630 + EA group showed an inflammatory response characterized with mucosal erosion, congestion, reduced or absent crypt and neutrophil infiltration. The TNBS + EA + DMSO group significantly improved the colonic lesions (Figures 4D,E).

**FIGURE 5:**
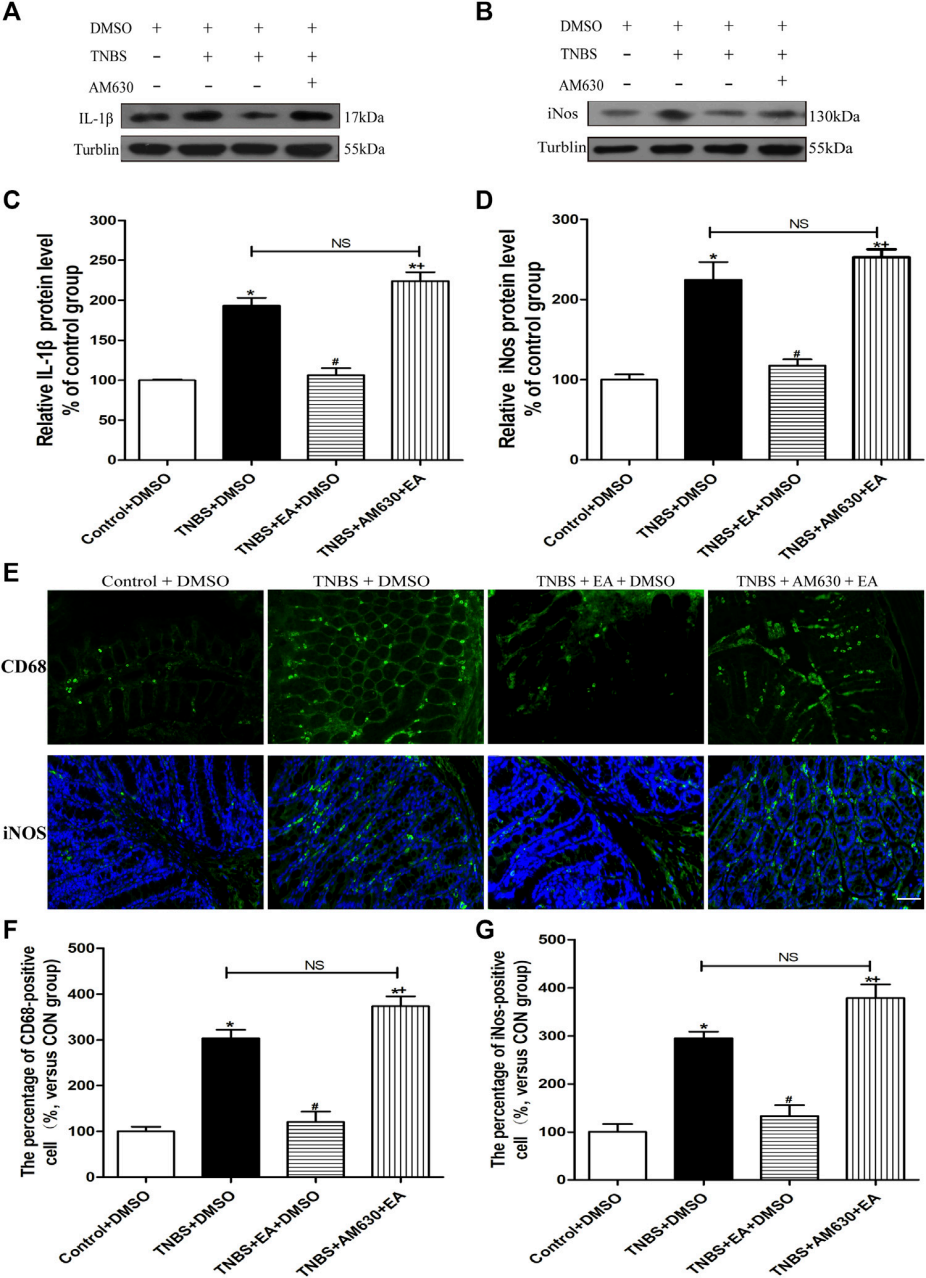
AM630 reverses the EA’s anti-inflammatory effects in TNBS-treated mice. Representative western blot images show the protein level of IL-1β **(A)** and iNOS **(B)** in the colon tissues. Summary data show the relative protein levels of IL-1β **(C)** and iNOS **(D)** in the colon tissues. **(E)** Representative images of immunofluorescence staining in which cell membrane were labeled with anti-CD68 and anti-iNOS in the colon tissues. Summary of the number of CD68^−^ **(F)** and iNOS- **(G)** positive cells per observation field. Data are expressed as mean ± SEM (*n* = 6 per group). Scale bar, 50 μm *p < 0.05 versus with Control + DMSO group, #*p* < 0.05 versus with TNBS + DMSO group, +*p* < 0.05 versus with TNBS + EA + DMSO group. NS, no significance versus TNBS + EA + AM630 group as indicated.

Treatment with AM630 antagonized the anti-inflammatory effect of EA (Figure 5). In the presence of AM630, EA failed to reduce the protein levels of IL-1β and iNOS (*p >* 0.05, Figures 5A–D) and the number of fluorescence-positive cells for iNOS and CD68 in the inflamed colon tissues by TNBS (*p >* 0.05, Figures 5E,F). These results suggest that the antinociceptive and anti-inflammatory effects of EA are mediated by the CB2 receptors.

### The Antinociceptive and Anti-Inflammatory Effects of EA on TNBS-Treated Mice Depend on CB2 Receptors

**FIGURE 6:**
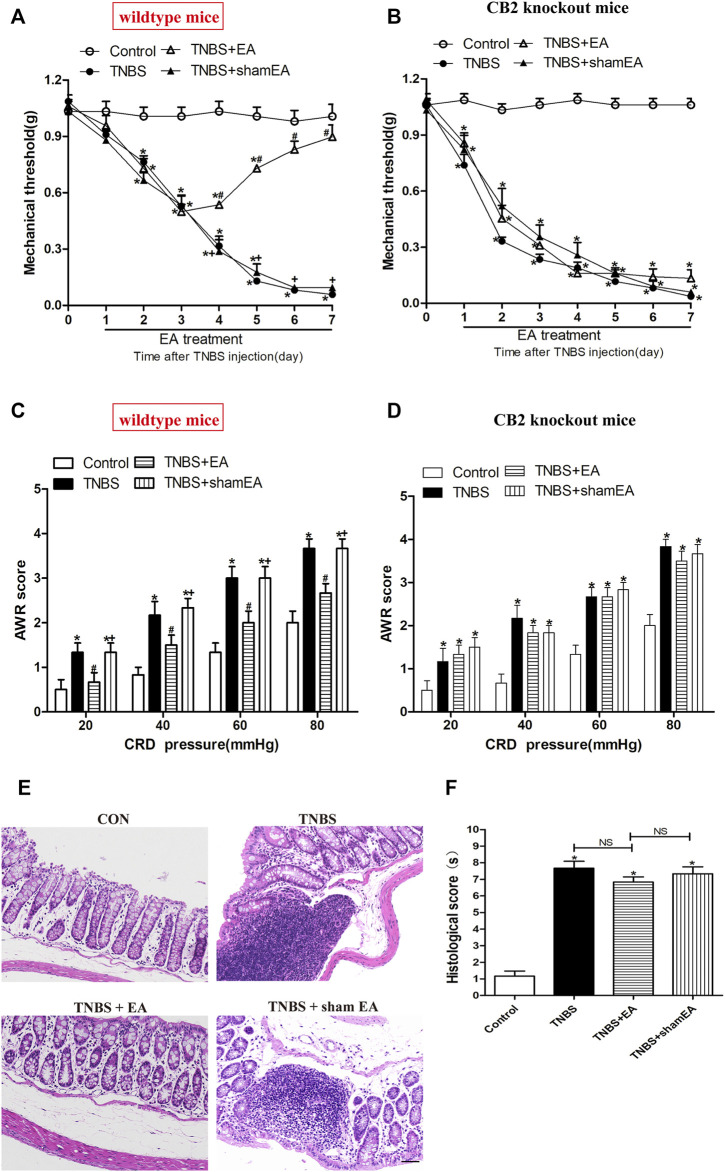
EA attenuates pain hypersensitivity induced by TNBS through CB2 receptors. **(A**–**D)** Time course of mechanical withdrawal threshold in wild-type **(A)** and CB2 receptor knockout **(B)** mice treated with TNBS. AWR scores in wild-type **(C)** and CB2 receptor knockout **(D)** mice treated with TNBS on day 7. **(E)** Representative H&E staining of the colon tissue sections in CB2 receptor knockout mice. Scale bar, 50 μm. **(F)** Histological score in each group (*n* = 6 per group). *p < 0.05 versus with Control group, #*p* < 0.05 versus with TNBS group, +*p* < 0.05 versus with TNBS + EA group. NS, no significance versus Sham EA group as indicated.

To validate the role of CB2 receptors in the therapeutic effect of EA on TNBS-induced colitis in mice, we tested the EA's effect in CB2 KO mice treated with TNBS. The visceral hyperalgesia and mechanical allodynia were present in wild-type and CB2 receptor KO mice after TNBS treatment. However, EA failed to reduce visceral hyperalgesia and mechanical allodynia in CB2 KO mice treated with TNBS (*p >* 0.05, Figures 6A–D). Also, treatment with EA had no significant effect on the histological inflammation and pathology of colitis in CB2 KO mice treated with TNBS, as evidenced by extensive destruction of tissue architecture, loss of intestinal crypts and goblet cells, marked mucosal erosion and congestion (*p >* 0.05, Figures 6E,F).

**FIGURE 7:**
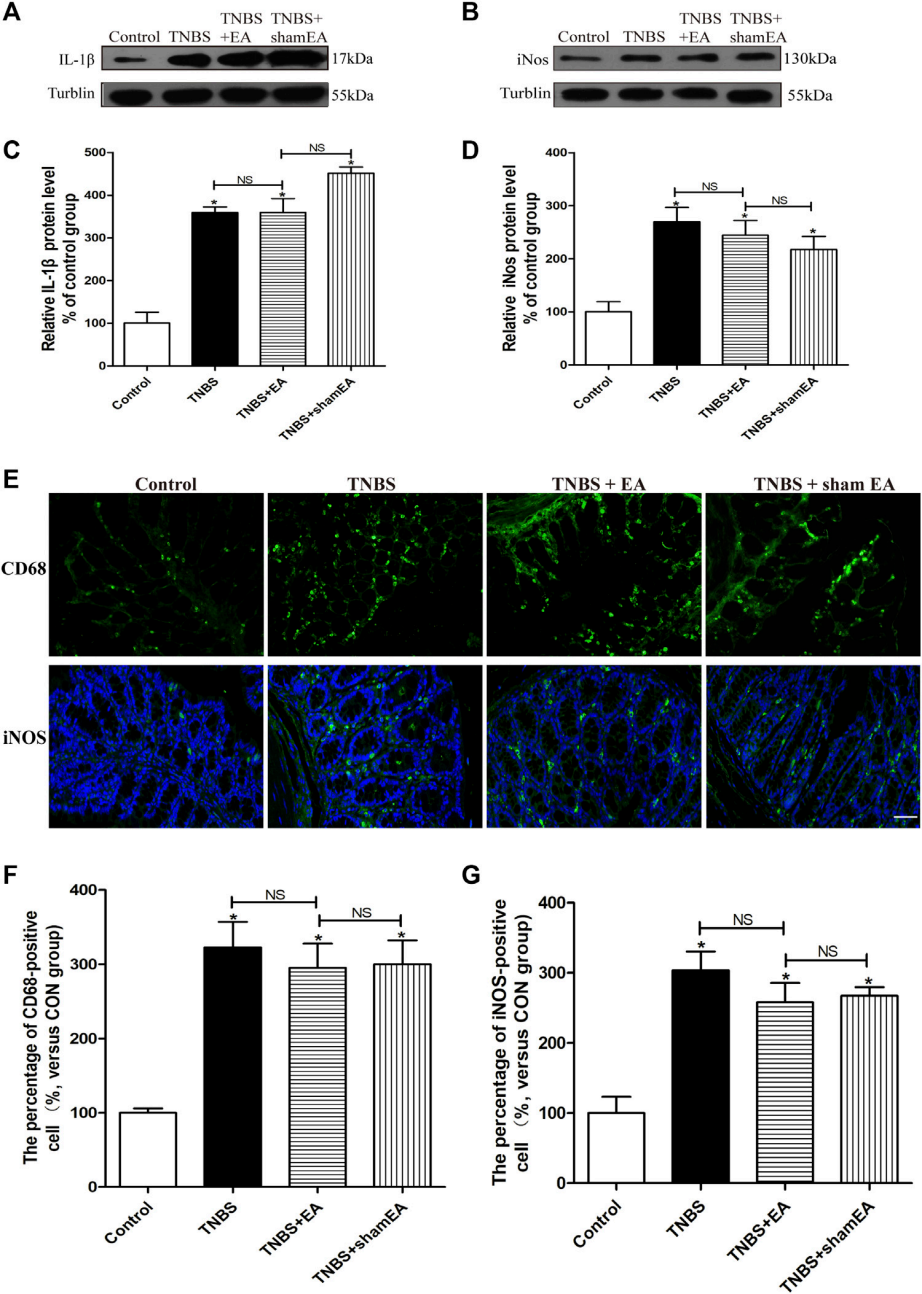
EA’s anti-inflammatory effects on TNBS-treated mice is mediated by CB2 receptors. **(A**–**D)** Representative western blot images show the protein level of IL-1β **(A)** and iNOS **(B)** in the colon tissues. Summary data show the relative protein levels of IL-1β **(C)** and iNOS **(D)** in the colon tissues. **(E)** Representative images of immunofluorescence labeled with anti-CD68 and anti-iNOS antibodies in the colon tissues. Summary data show the number of CD68^−^ **(F)** and iNOS- **(G)** positive cells per observation field. Data are expressed as mean ± SEM (*n* = 6 per group). Scale bar, 50 μm *p < 0.05 versus with Control group, #*p* < 0.05 versus with TNBS group, +*p* < 0.05 versus with TNBS + EA group. NS: no significance versus Sham EA group as indicated.

Similar to WT mice, TNBS significantly increased the protein levels of IL-1β mature fragments and iNOS and the number of iNOS- and CD68-positive cells in the colonic tissues in CB2 receptor KO mice (*p >* 0.05, Figures 7A–D). However, EA treatment failed to significantly reduce the elevated protein levels of IL-1β mature fragments and iNOS and the number of iNOS- and CD68-positive cells in the colonic tissues (*p >* 0.05, Figures 7A–D). These data suggest that CB2 receptors play a critical role in the antinociceptive and anti-inflammatory effects of EA in the mouse model of IBD.

## Discussion

Disorders of the intestinal immune system play a major role in the pathophysiology of IBD. The macrophages residing in the lamina propria maintain intestinal homeostasis under normal physiological conditions, but after inflammation or infection, the resident monocytes in the intestine and their derived pro-inflammatory macrophages enter the myenteric layer and further recruit more pro-inflammatory macrophages, thus further exacerbating IBD by releasing inflammatory factors (Grainger et al., 2017; Bain and Schridde, 2018). In the present study, we showed that only a small number of macrophages were present in the control group, which is consistent with the finding that in the normal intestine, only a few infiltrating immune cells are present (He et al., 2019). The increased abundance of macrophage expression after TNBS indicates that dysregulated immune/inflammatory responses on play a role in IBD. Importantly, we found that EA suppressed the newly recruited macrophages in the muscle layer, inhibited IL-1β and iNOS overexpression, and attenuated TNBS-induced visceral hypersensitivity. In addition, EA further increased the CB2 receptor expression on the macrophages in the colonic tissues. However, the CB2 receptor antagonist or CB2 receptor deletion antagonized the antinociceptive and anti-inflammatory effects of EA, suggesting that the beneficial effects of EA are mediated by CB2 receptors. Therefore, EA at BL25 points likely reduces TNBS-induced inflammation by activating CB2 receptors. EA may exert anti-inflammatory and antinociceptive effects by inhibiting the accumulation of newly recruited macrophages and reducing the levels of inflammatory cytokines. Collectively, these results suggest that CB2 receptors in the colonic tissues are a major component of the EA-produced anti-inflammatory and antinociceptive actions in the IBD model.

TNBS triggers visceral and somatic hypersensitivity after intracolonic injection (Viana-Cardoso et al., 2015). The onset and maintenance of colonic hypersensitivity may arise from the nociceptive inputs from the colon that sensitize the spinal dorsal horn neurons to cause somatic and visceral hypersensitivity (Zhou et al., 2008). This explanation is supported by the findings that the mechanical pain threshold after TNBS treatment begins to decrease significantly on the third day and reaches a peak on the fifth day. The rat colonic tissues recovered after 7 days of enema administration of 2,4-dinitrobenzene sulfonic acid (Lucarini et al., 2020). Similarly, we found that 7 days after TNBS treatment, histological analysis of the colon showed partial recovery of the mucosa, but a small amount of ulcerated areas were still seen with the loss of lining epithelium and infiltration of wall-permeable immune cells (mainly neutrophils, lymphocytes and macrophages), crypt abscesses, altered goblet cells and edema. Lv et al. reported that EA stimulation reduced the macroscopic pathologic changes and visceral hypersensitive of TNBS-induced colitis in rats (Lv et al., 2019). Similarly, our study also consistently found that EA alleviates visceral hyperalgesia, mechanical allodynia and intestinal pathology induced by TNBS. In a word, EA plays a key role in the recovery of IBD.

Resident macrophages that maintain intestinal homeostasis have a strong phagocytic effect but do not elicit a significant inflammatory response in mouse and human intestines (Bernardo et al., 2018; Bujko et al., 2018). When the homeostasis is disturbed, the composition of the human intestinal macrophage pool will change significantly. In the mucosa of patients with IBD, there is an accumulation of pro-inflammatory macrophages that produce large amounts of IL-1β, IL-6, TNF-α, reactive oxygen intermediates, and nitric oxide, making them different from the macrophages in the healthy intestine (Singh et al., 2016). A similar infiltration of pro-inflammatory monocytes and macrophages is seen in animal models of dextran sulphate sodium (DSS) (Gong et al., 2018; Mei et al., 2019). This infiltration is characterized by the reversal of the ratio of resident macrophages to proinflammatory macrophages, which is due to the accumulation of monocytes and their derived macrophages. Infiltrated macrophages showed typical pro-inflammatory features, including the production of TNF-α, IL-6, IL-1β production, and the expression of iNOS. Similarly, in our TNBS-treated model, intestinal homeostasis was disrupted, as reflected by macrophage activation, increased the expression of IL-1β and iNOS.

Neuron-glia-immune interaction in the DRG, spinal cord and enteric nervous system is now considered to play a key role in the development of visceral hypersensitivity, which is associated with visceral pain accompanying IBD (Qiao and Tiwari, 2020). Inflammatory mediators released by macrophages can directly activate and sensitize colon-innervating afferents, leading to an enhanced response to chemical and mechanical stimuli, known as visceral pain/hypersensitivity (Kwon et al., 2013; Domoto et al., 2021). Thus, inactivation of macrophages may be at least partially underlie the antinociceptive effects of EA. Our results indicated that EA inhibited macrophage activation, along with suppressing the increased expression of IL-1β and iNOS. Thus, EA may regulated immune function and visceral hypersensitive in the gastrointestinal tract by inhibiting macrophage activation.

In the gastrointestinal tract, the activated endogenous cannabinoid system is involved in regulating gut motility, intestinal secretion and epithelial permeability, and immune function through cannabinoid receptors present (Hasenoehrl et al., 2016; Perisetti et al., 2020). The antinociceptive effects of cannabinoid oil used in adolescent and young adult patients with IBD have been clinically demonstrated (Hoffenberg et al., 2019). However, the addictive side effects of cannabis have led to ongoing restrictions on the clinical use of cannabis for IBD. Anandamide (AEA) and 2-arachidonoylglycerol (2-AG) function as retrograde messengers in descending pain regulatory pathways, and they are transported to the peripheral terminals of the CNS and primary afferent neurons to inhibit neurotransmitter release from presynaptic terminals (Gondim et al., 2012; Kano, 2014). In our previous study, in a rat model of CFA-induced inflammatory pain, EA significantly increased the level of AEA in inflammatory skin tissues and produced antinociceptive effects through activation of peripheral CB2 receptors (Chen et al., 2009). Our study showed for the first time that EA exerts antinociceptive effects and inhibits activation of macrophages and suppresses the expression of mature IL-1β and iNOS through activating CB2 receptors in the colonic tissues. The CB2 receptor antagonist AM630 or CB2 receptor knockout blocked the inhibitory effects of EA on macrophages activation in the colon tissues. This is consistent with previous studies showing that activation of CB2 receptors ameliorates DSS-induced colitis by enhancing the inhibition of NLRP3 inflammasome activation in macrophages (Ke et al., 2016).

Therefore, it is reasonable assumption that EA at BL25 appears to reduce TNBS-induced IBD inflammation by increasing endogenous cannabinoids and then increasing CB2 receptors, thereby inhibits activation of macrophages and suppresses inflammatory cytokine levels and visceral hypersensitivity. Thus, CB2 receptor agonists could be used to treat IBD.

Although our findings have great potential for EA in the treatment of IBD patients, there are some limitations to this study. At present, this study is lack of EA for the determination of the content of other components of endocannabinoid system; In addition, other more effective acupoints for IBD should be explored.

## Conclusion

Our study shows that EA at BL25 is effective in reducing visceral pain in a mouse model of IBD. Our results further suggest that the antinociceptive effect of EA may be attributed to the CB2 receptor-mediated inhibition of macrophage activation and expression of IL-1β and iNOS. Our findings provide new information on the mechanism by which EA activates CB2 receptors to reduce visceral pain associated with IBD.

## Data Availability

The original contributions presented in the study are included in the article/Supplementary Material, further inquiries can be directed to the corresponding authors.
